# Determination of optimal cut-off points after a high-fat meal corresponding to fasting elevations of triglyceride and remnant cholesterol in Chinese subjects

**DOI:** 10.1186/s12944-019-1146-9

**Published:** 2019-11-25

**Authors:** Jin Xu, Yan-Qiao Chen, Shui-Ping Zhao, Ling Liu

**Affiliations:** 1 Department of Cardiovascular Medicine, The Second Xiangya Hospital, Central South University, #139 Middle Renmin Road, Changsha, Hunan 410011 People’s Republic of China; 2 Research Institute of Blood Lipid and Atherosclerosis, Central South University, Changsha, Hunan 410011 People’s Republic of China

**Keywords:** Postprandial, Non-fasting, Triglyceride, Remnant cholesterol, Chinese subjects

## Abstract

**Background:**

Postprandial high triglyceride (HTG), marking elevated level of remnant cholesterol (RC), is an independent risk factor of coronary heart disease (CHD). The postprandial cut-off points for HTG and high RC (HRC) after a daily meal are recommended as 2.0 mmol/L and 0.9 mmol/L, respectively, by the European Atherosclerosis Society (EAS), while those after a high-fat meal in Chinese subjects were not explored.

**Methods:**

Ninety subjects, including 60 CHD patients (CHD group) and 30 non-CHD controls (CON group), were enrolled in this study. Serum levels of blood lipids, including calculated RC, were monitored at 0, 2, 4 and 6 h after a high-fat meal with 800 kcal and 50 g fat. Analysis of c-statistic was used to determine the cut-off points for postprandial HTG and HRC.

**Results:**

Postprandial levels of triglyceride (TG) and RC significantly increased and peaked at 4 h after a high-fat meal in two groups, although those in CHD group were significantly higher (*P* < 0.05). The optimal cut-off point to predict HTG at 4 h corresponding to fasting TG ≥ 1.7 mmol/L was 3.12 mmol/L, and that to predict HRC at 4 h corresponding to fasting RC ≥ 0.8 mmol/L was 1.36 mmol/L. According to the new cut-off points, the omissive diagnosis rates of postprandial HTG and HRC decreased obviously.

**Conclusion:**

The cut-off points of postprandial HTG and HRC in Chinese subjects after a high-fat meal were higher than those after a daily meal recommended by the EAS, indicating that specific cut-off points should be determined after a certain high-fat meal.

## Introduction

According to 2016 Chinese guideline for the management of dyslipidemia in adults, appropriate triglyceride (TG) level is defined as fasting TG <1.7 mmol/L, borderline elevation as 1.7 mmol/L ≤ fasting TG <2.3 mmol/L, and elevation as fasting TG ≥2.3 mmol/L [1]. Actually, non-fasting or postprandial high TG (HTG) is also an independent risk factor of coronary heart disease (CHD) [2–4]. Non-fasting levels of blood lipids can be detected at anytime within 8 h after a daily meal according to individual’s dietary habits or at appointed time-points after a high-fat meal with specified content. The former is similar to a random blood glucose measurement while the latter is analogue to oral glucose tolerance test [5]. According to the European joint consensus statement from the European Atherosclerosis Society (EAS) and the statement from the American Heart Association (AHA), non-fasting TG levels in patients whose fasting TG < 1.7 mmol/L would not be expected to raise above 2.0 mmol/L (175 mg/dL) and 2.26 mmol/L (200 mg/dL), respectively, after consuming a daily low fat meal before blood sampling [6, 7]. However, the cut-off point to diagnose non-fasting HTG is 1.98 mmol/L (175 mg/dL) in middle aged and older apparently healthy American women basing on the Women’s Health Study [8], which is very close to the optimal non-fasting TG cut-off point (2.0 mmol/L) in Japanese employees [9]. Until now, similar expert consensus or study is still rare in China. Recently, we determined a cut-off point for non-fasting TG level 2.02 mmol/L at 4 h after a daily breakfast in relation to fasting TG ≥ 1.7 mmol/L in 109 Chinese subjects with and without overweight [10], which is very close to the values recommended by the European joint consensus statement or reported by other scholars [6, 8, 9]. HTG indicates the increases in circulating triglyceride-rich lipoproteins (TRLs) and their remnants, remnant lipoproteins [4, 11, 12]. The latter is as atherogenic as low-density lipoprotein (LDL) [13, 14]. Level of remnant lipoprotein cholesterol (i.e. remnant cholesterol, RC) can be measured directly or estimated by a certain formula [15]. Numerous studies showed that estimated RC independently predicted the risk of CHD [16–18]. It is recommended that non-fasting RC level should not be above 0.9 mmol/L (35 mg/dL) after a daily meal for individuals with fasting RC < 0.8 mmol/L (30 mg/dL) by the EAS [6], however, there is no similar recommendation in China.

Comparing with above studies about non-fasting cut-off point for TG elevation after a daily meal [7–9], those after a high-fat meal was scarce although fat tolerance test has been employed to evaluate postprandial HTG for a longer time since last century [19]. Only Mexican scholars had identified an optimal TG truncation of 3.17 mmol/L (280 mg/dL) after a high-fat meal with 960 kcal through receiver operating characteristic (ROC) curve analysis [20]. We previously established an oral fat tolerance test with 800 kcal and 56% fat content according to Chinese dietary habits, and observed the different changes in non-fasting TG levels between CHD patients and controls [21–23]. However, there was no study about non-fasting cut-off point for TG or RC elevation after a high-fat meal in Chinese yet.

In this study, we compared the changes in levels of TG and estimated RC between CHD patients and their controls after a high-fat meal, aimed to identify optimal non-fasting cut-off points of TG and RC in Chinese individuals after a high-fat meal in relation to fasting TG ≥ 1.7 mmol/L and RC ≥ 0.8 mmol/L, respectively.

## Methods

### Study subjects

Ninety subjects, including 60 documented CHD patients (CHD group) and 30 non-CHD controls (CON group), were recruited in this study in the Department of Cardiovascular Medicine of the Second Xiangya Hospital, Central South University. CHD was defined as a history of myocardial infarction and/or angiographically proven coronary atherosclerosis in patients with angina pectoris. Contemporaneous controls who had no clinical history and manifestation of CHD were classified into CON group [24].

All subjects were invited to filled out a questionnaire on medical history and use of medication before participant. No subjects had a history of diabetes, thyroid diseases, liver and kidney diseases, autoimmune disease, cancer or other severe medical illnesses, and no one took oral hypoglycaemic or hypolipidemic agents. The study was approved by the Ethics Committee of the Second Xiangya Hospital of Central South University and informed consent was gained from all participants.

### Oral high-fat tolerance test

After at least 12 h of overnight fasting, all subjects were given a high-fat meal. The oral high-fat tolerance test was undertaken as described previously by a nutritionist [23]. The high-fat meal consisting of 800 kcal with 50 g of fat (345 mg of cholesterol), 28 g of protein, and 60 g of carbohydrate was given in Chinese traditional form, including vegetable blending oil, noodles, egg and pork floss. All subjects were requested to finish the meal in 15 min. During 6-h test, subjects were allowed to drink only water and prohibited to smoke, drink wine or eat any food. Strenuous exercises were not recommended, and only slow walking was allowed.

### Laboratory assays

Blood samples were taken before and at 2, 4 and 6 h after the high-fat meal. All blood samples were centrifuged at 4 °C 3000 rpm for 15 min. Serum levels of total cholesterol (TC) and TG were measured by automated enzymatic assays, and that of high-density lipoprotein cholesterol (HDL-C) were measured by a commercially available direct method, on a HITACHI 7170A analyzer (Instrument Hitachi Ltd., Tokyo, Japan) by a laboratory technician who had no idea of this study. LDL-cholesterol (LDL-C) level was calculated using the Friedewald formula: LDL-C = TC - (HDL-C) - (TG/2.2) when TG was < 4.5 mmol/L, otherwise it was directly measured by chemical masking method. RC level was estimated by the following formula, RC = TC - (HDL-C) - (LDL-C). Non-HDL-C = TC - (HDL-C). Fasting glucose level was measured using the glucose oxidase method.

### Statistical analysis

Quantitative variables were expressed as mean ± standard deviation (SD) unless were specifically explained, and qualitative variables were expressed as numbers and percentages. Differences between the intra- and intergroup means were analyzed by unpaired *t*-test or *one-way* analysis of variance. Categorical variables were compared using *chi-squared* statistic tests. The area under the curve (AUC) and the increment of AUC (iAUC), representing the increase in area after a high-fat meal above fasting levels of TG and RC, were estimated by trapezoid method. The optimal cut-off points for non-fasting TG and RC levels at 4 h were determined using ROC curve analysis. The rate of omissive diagnosis was defined as the percentage of normal fasting TG or RC in subjects with postprandial HTG or high RC (HRC). All statistical analyses were performed with SPSS version 25.0. All *P* values were 2-tailed, and *P* < 0.05 was considered statistically significant.

## Results

### Clinical characteristics and fasting blood lipids of two groups

There were no significant difference in age, gender, body mass index (BMI), systolic or diastolic blood pressure, heart rate, percentage of overweight or smoking, and fasting glucose level between two groups. Levels of fasting TG, TC, LDL-C, non-HDL-C and RC were significantly higher while fasting HDL-C level was significantly lower in CHD group (*P*<0.05, Table 1). Fasting TG elevation (≥1.7 mmol/L) was found in 42 (70%) CHD patients and 10 (33.3%) controls, which was similar to that of fasting RC elevation (≥0.8 mmol/L) in each group (CHD: 68.3%; CON: 30%).

**Table 1:** Comparison of clinical features between two groups

	CHD (*n* = 60)	CON (*n* = 30)	*P* value
Age (y)	52.22 ± 7.27	49.40 ± 11.25	NS
Gender (M/F)	42/18	21/9	NS
BMI (Kg/m^2^)	24.47 ± 2.26	23.91 ± 2.75	NS
Overweight [n(%)]	35 (58.33)	12 (40.00)	NS
Systolic pressure (mmHg)	123.68 ± 15.18	120.43 ± 16.92	NS
Diastolic pressure (mmHg)	80.69 ± 9.36	78.48 ± 10.92	NS
Heart rate (bpm)	77.12 ± 8.13	77.12 ± 9.34	NS
Current smoking [n(%)]	23 (38.33)	8 (26.67)	NS
Fasting glucose (mmol/L)	5.22 ± 0.69	4.99 ± 0.84	NS
TG^a^ (mmol/L)	2.36 ± 1.20	1.41 ± 0.57	< 0.001
TC (mmol/L)	5.16 ± 0.78	4.44 ± 0.77	< 0.001
HDL-C^a^ (mmol/L)	1.12 ± 0.19	1.26 ± 0.31	< 0.05
LDL-C (mmol/L)	2.98 ± 0.65	2.54 ± 0.69	< 0.01
non-HDL-C (mmol/L)	4.04 ± 0.74	3.18 ± 0.71	< 0.001
RC^a^ (mmol/L)	1.06 ± 0.53	0.64 ± 0.25	< 0.001

*BMI* Body mass index, *bpm* Beats per minute, *TG* Triglyceride, *TC* Total cholesterol, *HDL-C* High-density lipoprotein cholesterol, *LDL-C* Low-density lipoprotein cholesterol, *non-HDL-C* Non high-density lipoprotein cholesterol, *RC* Remnant lipoprotein cholesterol, *NS* No significance

^a^ logarithmic transformation for non-normal distribution

### Postprandial changes in serum levels of blood lipids in two groups

**Fig. 1:**
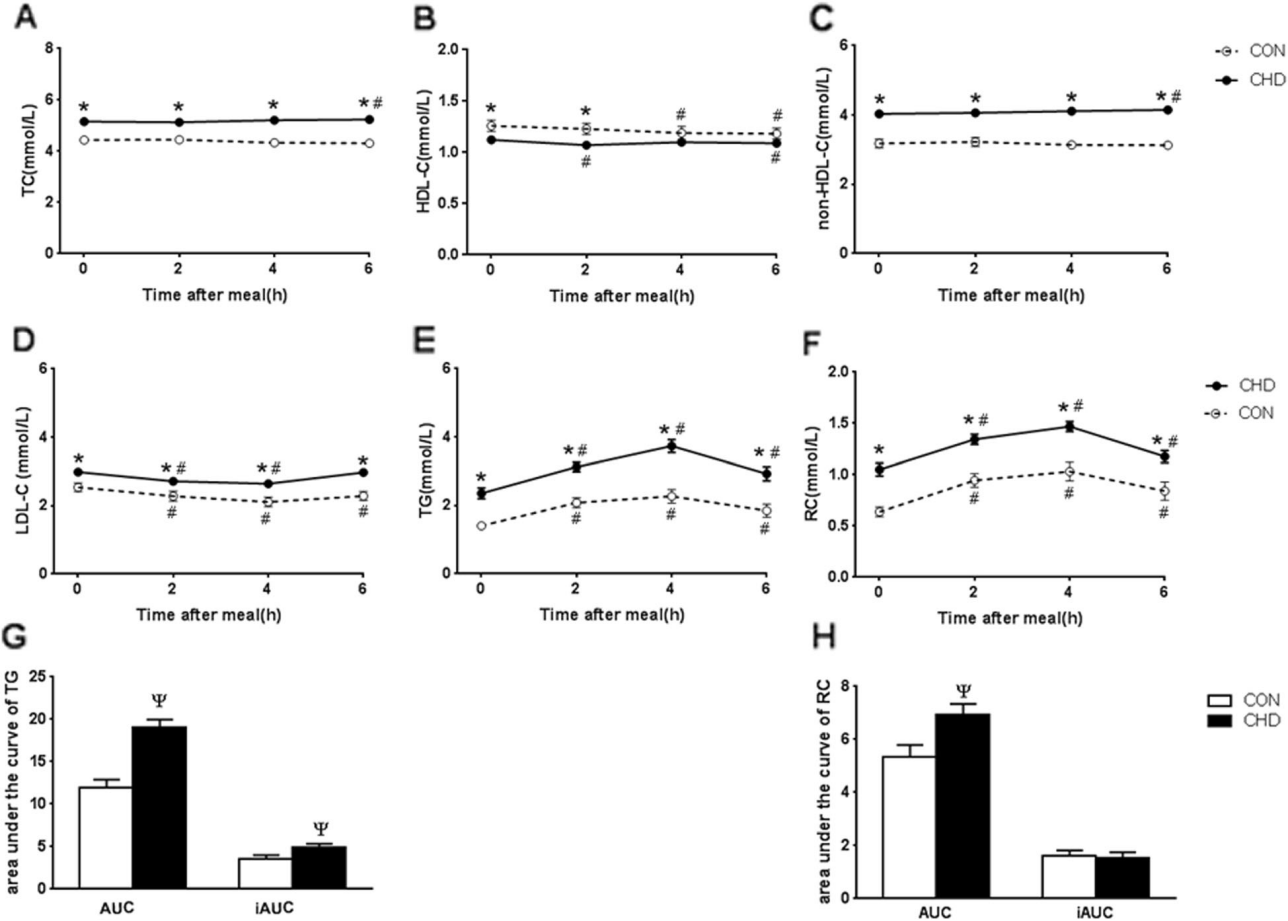
Changes in serum levels of blood lipids after a high-fat meal in two groups. **a**-**f** Postprandial changes in serum levels of TC, HDL-C, non-HDL-C, LDL-C, TG, and RC after a high-fat meal in two groups. **g** and **h** Comparisons of AUC and iAUC of postprandial TG or RC level after a high-fat meal between two groups. The bar represent standard error of the mean. * *P* < 0.05 when compared with CON group at the same time point. ^#^ *P* < 0.05 when compared with fasting level in the same group. Ψ *P* < 0.05 when compared with CON group

Significant differences in blood lipids between CHD group and CON group were showed at most time points after a high-fat meal (*P*<0.05, Fig. 1a-f).

Although postprandial changes in levels of TC, HDL-C and non-HDL-C were slight (Fig. 1a-c), postprandial decrease in LDL-C level was significant at 2 h and 4 h in both groups (*P*<0.05, Fig. 1d). Postprandial LDL-C level at 6 h in CON group was still significantly lower than its fasting value (*P*<0.05) while that in CHD group restored to near its fasting value (Fig. 1d).

Both TG and RC levels increased tremendously at all postprandial time points (*P* < 0.05) and peaked at 4 h after a high-fat meal in both groups (Fig. 1e & f). AUC of TG or RC in CHD group was significantly higher than that in CON group (*P* < 0.05), however, only iAUC of TG in CHD group was significantly higher than that in CON group, but not RC(*P* < 0.05, Fig. 1g & h).

### Comparisons of percentages of postprandial dyslipidemia according to cut-off points recommended by the EAS between two groups

**Fig. 2:**
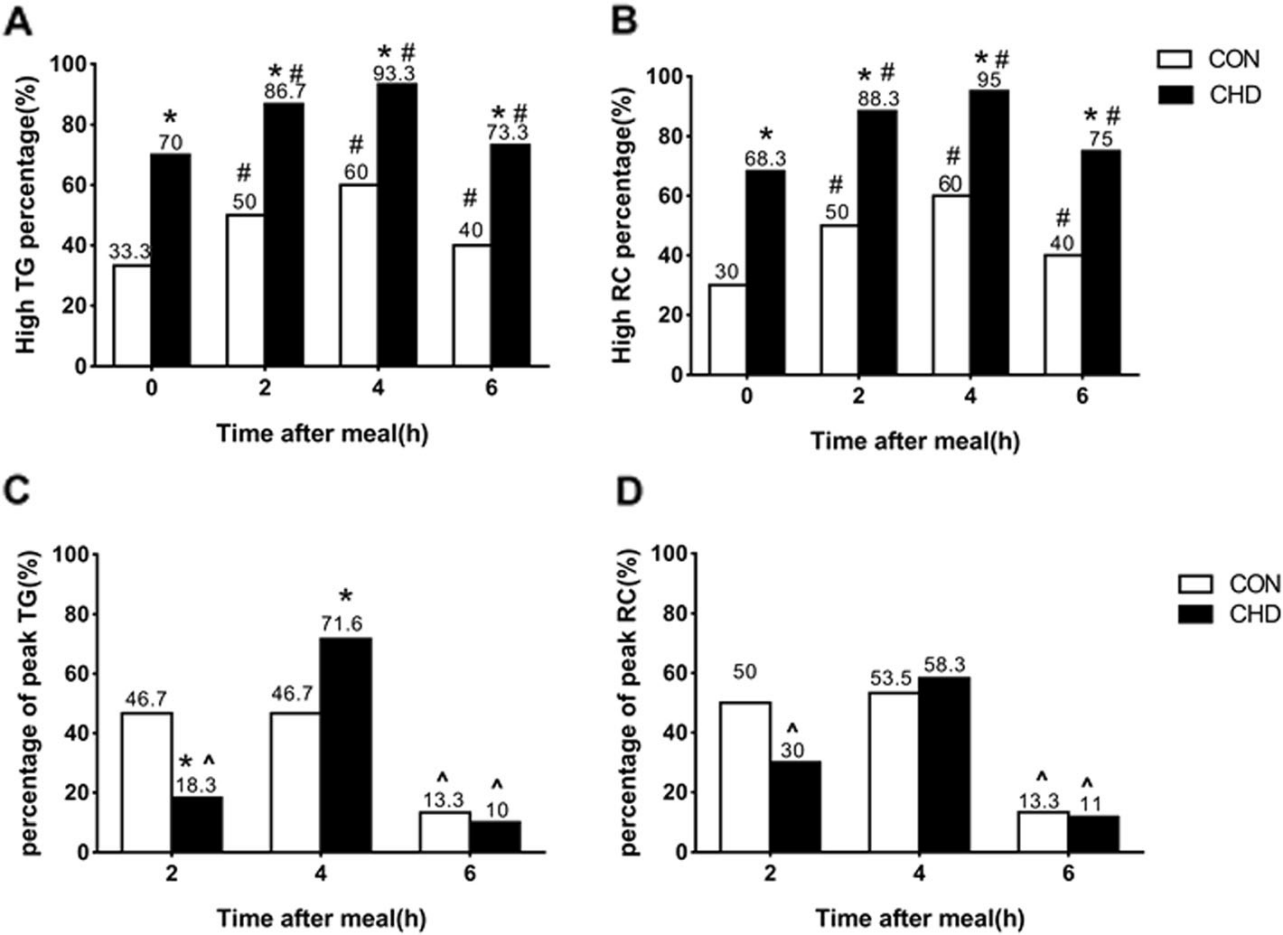
Comparisons of the fasting and postprandial percentages of HTG and HRC between two groups according to the criteria recommended by the European Atherosclerosis Society (EAS) after a daily meal. **a** and **b** The percentages of HTG or HRC according to fasting and postprandially TG or RC level, respectively. **c** and **d** Comparisons of percentages of subjects reaching the peak value of TG or RC level at different time point after a high-fat meal. * *P* < 0.05 when compared with CON group at the same time point. ^#^ *P* < 0.05 when compared with the value in fasting state in the same group. ^∧^ *P* < 0.05 when compared with the postprandial percentage at 4 h in the same group

Postprandial levels of TG and RC in this study were initially evaluated according to the non-fasting cut-off points after a daily meal recommended by the EAS. Postprandial percentages of HTG and HRC in CHD group were prominently higher than those in CON group, respectively (*P*<0.05). Percentage of HTG or HRC in postprandial state at any time point was enormously higher than that in fasting state in each group (*P*<0.05). The highest percentage of HTG or HRC in each group was found at 4 h, which was more than 90% in CHD group and reached 60% in CON group (Fig. 2a & b).

Furthermore, when the time of peak level of TG or RC was analyzed, it was found mainly at 4 h in CHD group but nearly equally at 2 h and 4 h in CON group. There was significant difference in percentage of TG reaching the peak level at 2 h or 4 h between two groups (*P*<0.05, Fig. 2c & d).

### Comparisons of percentages of postprandial dyslipidemia according to new cut-off points determined by ROC curve analysis between two groups

**Fig. 3:**
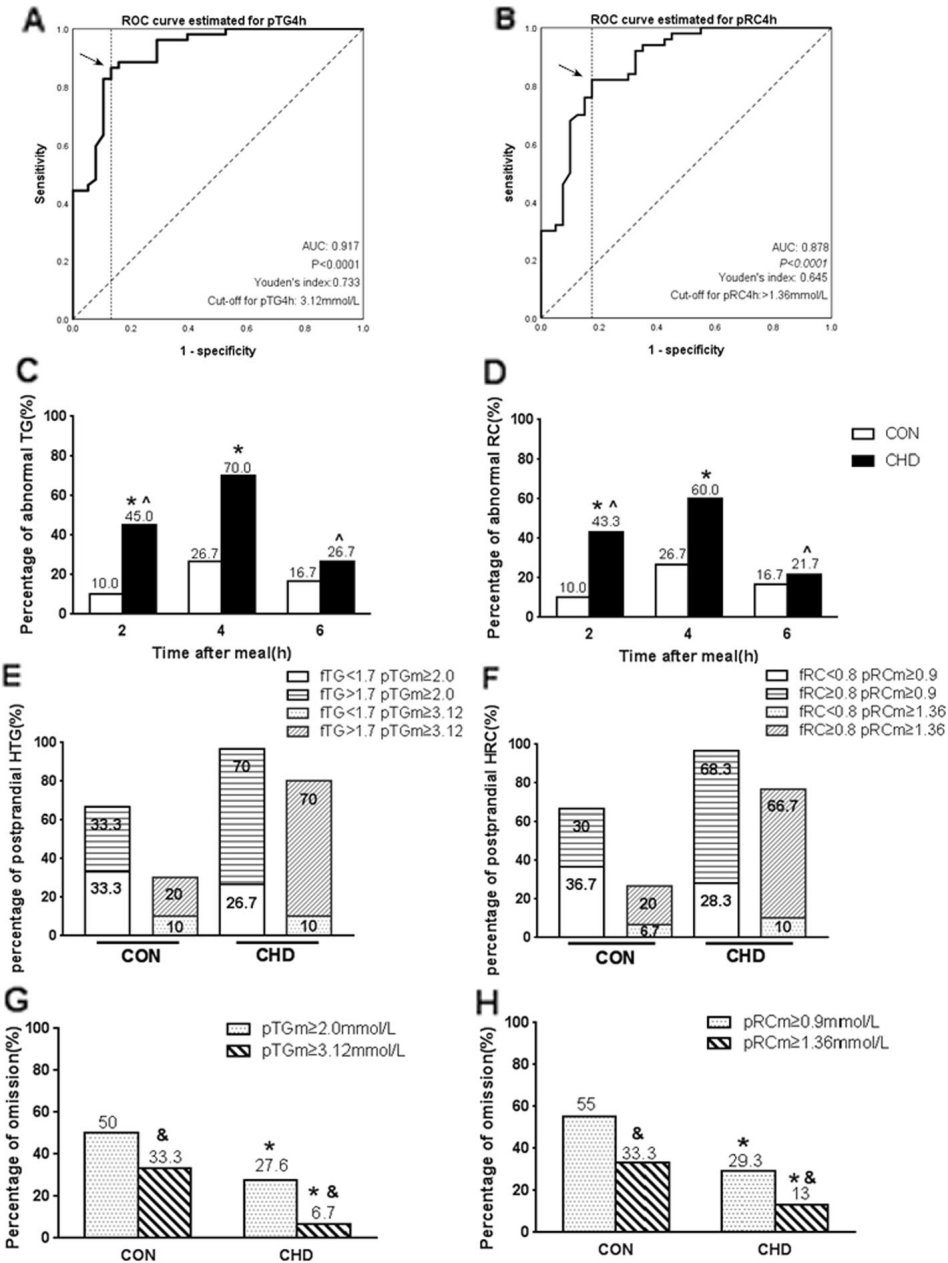
Comparisons of the fasting and postprandial percentages of HTG and HRC between two groups according to the cut-off points via ROC analysis after a high-fat meal. **a** and **b** ROC analysis and Youden’s index determined a cut-off point for postprandial TG or RC level at 4 h (pTG4h or pRC4h) after a high-fat meal, the cut-off point was indicated by the solid arrow. **c** and **d** Comparisons of postprandial percentages of HTG or HRC at different time point according to new cut-off points. **e** and **f** Comparison of percentage of postprandial HTG or HRC according to different postprandial criteria in two group. **g** and **h** Comparison of omissive diagnosis rates according to different postprandial criteria of HTG and HRC between two groups. * *P* < 0.05 when compared with CON group according to a same criteria. ^∧^ *P* < 0.05 when compared with the percentage at 4 h in the same group. ^&^ *P* < 0.05 when compared with omissive diagnosis rates according to EAS criteria in the same group

ROC curve analysis was performed and Youden’s index was calculated according to the sensitivity and specificity of each possible cut-off point in the statistical results. The optimal cut-off point for TG at 4 h to predict HTG in relation to fasting TG ≥ 1.7 mmol/L was 3.12 mmol/L (sensitivity 86.5%, specificity 86.8%, and AUC 0.917), and that for RC at 4 h to predict HRC in relation to fasting RC ≥ 0.8 mmol/L was 1.36 mmol/L (sensitivity 82%, specificity 82.5%, and AUC 0.878) (Fig. 3a & b).

According to the new cut-off points, the percentages of postprandial HTG and HRC decreased obviously. They were no more than 70% in CHD group while less than 30% in CON group. Significant difference in percentage of postprandial HTG or HRC between two groups was found at 2 h and 4 h (*P*<0.05). Furthermore, the highest percentage of postprandial hyperlipidemia, i.e. HTG or HRC, was still found at 4 h in both groups (*P*<0.05, Fig. 3c & d).

When postprandial HTG or HRC was defined as the maximal postprandial TG (pTGm) ≥ 2.0 mmol/L or RC (pRCm) ≥ 0.9 mmol/L, postprandial HTG or HRC was found in about two-thirds controls and near 100% patients with CHD no matter fasting TG or RC level was borderline-high or not. When postprandial HTG or HRC was defined as pTGm ≥3.12 mmol/L or pRCm ≥1.36 mmol/L, the percentage of postprandial HTG or HRC obviously decreased in each group, which mainly occurred in the subjects with appropriate fasting TG or RC level, while that changed mildly in those with fasting HTG or HRC, especially in CHD group (Fig. 3e & f).

For subjects with appropriate fasting TG or RC level, the diagnosis of postprandial HTG or HRC could be omitted without detecting of non-fasting level after a high-fat meal. When the EAS cut-off points of postprandial hyperlipidemia were utilized, the omissive diagnosis rates of postprandial HTG and HRC were near 30% in CHD group and about 50% in CON group, respectively. When the new cut-off points were utilized, they obviously decreased to ≤13% in CHD group and 33.3% in CON group (Fig. 3g & h).

## Discussion

In this study, prominent non-fasting hyperlipidemia, presenting as elevated levels of TG and RC, was found in CHD patients after a high-fat meal, especially at 4 h. Moreover, non-fasting cut-off points of HTG and HRC after a high-fat meal were firstly determined by ROC curve analysis in Chinese subjects, and they were obviously higher than those after a daily meal recommended by European consensus statement [6], however, the cut-off point of non-fasting HTG in our study was very similar to that in another Mexican research [20]. These results not only supported increased synthesis and/or reduced elimination of TRLs and their remnants in Chinese CHD patients after a high-fat meal, but also indicated the difference in non-fasting cut-off points of HTG and HRC between a high-fat meal and a daily meal.

Postprandial state plays a critical role in atherogenesis [25–27], which was supported by our finding that AUC of TG or RC in CHD group was significantly higher than that in CON group, although there was no significant difference in iAUC of RC between two groups. When compared with iAUC that represents postprandial increment of TG or RC after a high-fat meal, AUC seems to be a better index to reflect the sustained stimulus and even damages of TRLs and their remnants to artery walls during postprandial period, because it not only reflects the fasting levels of blood lipids but also covers their non-fasting increments and duration after a high-fat meal. However, non-fasting changes in TC, HDL-C and non-HDL-C levels seemed to be negligible when compared with those in TG and RC after a high-fat meal, although postprandial LDL-C level showed a significant decrease in this study. In fact, it is possible that the change of LDL-C could become insignificant with the increase in samples size [28].

For there were no recommendation about cut-off points of postprandial HTG and HRC in Chinese subjects, postprandial TG and RC elevation in this study were initially evaluated according to the non-fasting cut-off points after a daily meal recommended by the EAS [6]. Non-fasting HTG was found in 73.3–93.3% CHD patients and about 50% in the controls. The percentages of non-fasting HRC in two groups were very similar to those of non-fasting HTG because RC was estimated by formula. Under this condition, the proportion of so-called postprandial hyperlipidemia was too excessive to differentiate postprandial TRL metabolic abnormalities, especially in CHD patients, from physiological TG reaction to a high-fat meal, mainly in the controls [29]. We previously observed that postprandial TG elevation after a high-fat meal was greater than that after a low-fat meal in Chinese subjects [22], suggesting that it would be inappropriate to evaluate non-fasting levels of TG and RC in Chinese subjects after a high-fat meal according to the cut-off points after a daily meal recommended by the EAS .

In our study, the peak levels of TG and RC in CHD group predominantly emerged at 4 h in this study, while those in CON group were approximately in uniform distribution at 2 h and 4 h, indicating abnormal anabolism and/or catabolism of TRLs and their remnants in CHD patients. In view of a majority of subjects reaching the peak level of either TG or RC at 4 h after a high-fat meal, along with our previous finding that the optimal time point to evaluate postprandial TG elevation could be at 4 h after a high-fat meal with 800 kcal in Chinese subjects [30], we analyzed the cut-off points of non-fasting TG and RC elevation at this time point in the subsequent ROC curve analysis.

ROC curve analysis had been employed to determine optimal cut-off points for non-fasting high TG not only after a daily meal in the Women’s Health Study [8], but also after a high-fat meal in another study in Mexican adults [20]. In this study, the cut-off point of postprandial high TG was 3.12 mmol/L after a high-fat meal with 800 kcal in Chinese subjects, obviously higher than 2.0 mmol/L that is recommended by the EAS after a daily meal [6], while was very close to another cut-off point of TG, 3.16 mmol/L, after a high-fat meal with 960 kcal in Mexican adults [20]. The similarity between two cut-off points, 3.12 mmol/L and 3.16 mmol/L, for non-fasting high TG might not be simply attributed to a coincidence, but result from similar fat contents, 50 g vs. 52 g, of two high-fat meals in our study and the Mexican study. It suggests that high-fat meals with similar total calorie and fat content may share similar cut-off points, although the race of the subjects, dietary habits, time points for lipid testing and statistical methods were different between two studies.

Besides TG, we also analyzed RC in the postprandial state through ROC curve, as RC is an independent risk factor for cardiovascular diseases [13, 14]. A cut-off point of RC, 1.36 mmol/L, was determined to discriminate fasting RC elevation and much higher than that recommended by the EAS, 0.9 mmol/L. It indicated that for Chinese patients taking a high-fat meal with 800 kcal, a specific cut-off point of RC, but not that one after a daily meal [6], was needed.

When the new cut-off points determined by ROC curve analysis were used for evaluation, the percentage of postprandial HTG or HRC in each group was relatively lower than that evaluated by the cut-off points recommended by the EAS, however, the difference in multiples of the proportion of postprandial HTG or HRC between two groups increased obviously at 2 h and 4 h. For example, the proportion of postprandial HTG in CHD group was more than two times at 4 h as much as that in CON group, which was very similar to the condition in the fasting state. It indicated new cut-off points could be more helpful to differentiate the controls from CHD patients. Indeed, there were some individuals in CON group with risk factors, such as smoking [31, 32], overweight [33] and fasting TG or RC elevation [4], thus it was acceptable that HTG was found in 26.7% of the controls at 4 h after a high-fat meal.

It was worth noting that postprandial HTG or HRC was found in part of subjects with appropriate fasting TG or RC level when the maximal postprandial TG or RC level were evaluated, indicating that quite a part of subjects would be missed if postprandial blood lipids were not assessed. For this reason, omission diagnostic rates were analyzed according to different cut-off points. Interestingly, the omission diagnostic rates of postprandial HTG and HRC determined according to the new cut-off points were lower than that according to the EAS cut-off points in each group, suggesting that it is necessary to determine specific diagnose thresholds of postprandial hyperlipidemia for different population after a certain high-fat meal.

This study is associated with several limitations. First, the sample size in this study was small. Second, both LDL-C and RC were calculated by formulae, which may cause deviation with those directly measured [34].

## Conclusion

In conclusion, the cut-off points of postprandial HTG and HRC corresponding to fasting HTG and HRC were determined firstly in Chinese subjects after a high-fat meal and were found to be higher than those recommended by the EAS after a daily meal.

## Data Availability

The datasets analyzed during the current study are available from the corresponding author on reasonable request.

## Abbreviations

AHA: American Heart Association
AUC: Area under the curve
CHD: Coronary heart disease
CON: Non-CHD controls
EAS: European Atherosclerosis Society
HDL-C: High-density lipoprotein cholesterol
HRC: High remnant cholesterol
HTG: High triglyceride
iAUC: Increment of AUC
LDL: Low-density lipoprotein
LDL-C: Low-density lipoprotein cholesterol
Non-HDL-C: Non-high-density lipoprotein cholesterol
pRC: Postprandial RC
pRC4h: Postprandial RC at 4 h after meal
pRCm: The maximal postprandial RC
pTG: Postprandial TG
pTG4h: Postprandial TG at 4 h after meal
pTGm: The maximal postprandial TG
RC: Remnant cholesterol
ROC curve: Receiver operating characteristic
TC: Total cholesterol
TG: Triglyceride
TRLs: Triglyceride-rich lipoproteins
